# Construction of Ultrathin BiVO_4_‐Au‐Cu_2_O Nanosheets with Multiple Charge Transfer Paths for Effective Visible‐Light‐Driven Photocatalytic Degradation of Tetracycline

**DOI:** 10.1002/smtd.202301804

**Published:** 2024-06-10

**Authors:** Chen Wang, Amir Mirzaei, Yong Wang, Mohamed Chaker, Qingzhe Zhang, Dongling Ma

**Affiliations:** ^1^ Institut National de la Recherche Scientifique (INRS) Centre Énergie Materiaux et Télécommunications 1650 Boulevard Lionel‐Boulet Varennes Québec J3X1P7 Canada; ^2^ Shandong Key Laboratory of Environmental Processes and Health School of Environmental Science and Engineering Shandong University Qingdao 266237 China; ^3^ Shenzhen Research Institute of Shandong University Shenzhen 518057 China

**Keywords:** BiVO_4_ nanosheets, local surface plasmon resonance, multiple charge transfer paths, photocatalysis, tetracycline degradation, water treatment, Z‐scheme heterojunction

## Abstract

In this study, unique BiVO_4_‐Au‐Cu_2_O nanosheets (NSs) are well designed and multiple charge transfer paths are consequently constructed. The X‐ray photoelectron spectroscopy measurement during a light off‐on‐off cycle and redox capability tests of the photo‐generated charge carriers confirmed the formation of Z‐scheme heterojunction, which can facilitate the charge carrier separation and transfer and maintain the original strong redox potentials of the respective component in the heterojunction. The ultrathin 2D structure of the BiVO_4_ NSs provided sufficient surface area for the photocatalytic reaction. The local surface plasmon resonance (LSPR) effect of the electron mediator, Au NPs, enhanced the light absorption and promoted the excitation of hot electrons. The multiple charge transfer paths effectively promoted the separation and transfer of the charge carrier. The synergism of the abovementioned properties endowed the BiVO_4_‐Au‐Cu_2_O NSs with satisfactory photocatalytic activity in the degradation of tetracycline (Tc) with a removal rate of ≈80% within 30 min under visible light irradiation. The degradation products during the photocatalysis are confirmed by using ultra‐high performance liquid chromatography‐mass spectrometry and the plausible degradation pathways of Tc are consequently proposed. This work paves a strategy for developing highly efficient visible‐light‐driven photocatalysts with multiple charge transfer paths for removing organic contaminants in water.

## Graphical abstract

Multiple charge transfer paths, including the Z‐type based on the BiVO_4_/Cu_2_O Z‐scheme heterojunction, are constructed in the BiVO_4_‐Au‐Cu_2_O NSs to promote the charge separation. The ultrathin 2D structure of the BiVO_4 _NSs provided sufficient surface area for reaction. The LSPR effect of the Au NPs further enhanced the light absorption and promoted the excitation and transfer of hot electrons.

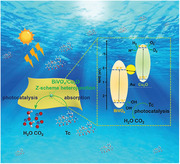

## 1 Introduction

The pharmaceutical residues of antibiotics with recalcitrant nature and persistent chemical structures have caused public concerns on human health and the environment in the past decades.^[^ ^1^ ^]^ Various strategies, including biological treatment, physical absorption, and chemical oxidation methods, were consequently explored for the removal of antibiotics in wastewater.^[^ ^2^ ^]^ Compared to the conventional biological treatment and physical absorption strategies, the advanced chemical oxidation technology shows specific advantages, such as rapid reaction rates and easy operation.^[^ ^3^, ^4^ ^]^ Photocatalysis has stood out as a promising strategy among the explored chemical oxidation methods to remove recalcitrant antibiotics via directly utilizing solar energy.^[^ ^5^ ^]^ Ideally, the persistent antibiotics, even trace contaminants in water, can be effectively decomposed and mineralized by photocatalysis without producing any secondary pollution.^[^ ^6^ ^]^ The selection and rational design of appropriate effective photocatalysts are hence critical and remain the core of photocatalysis.^[^ ^7^ ^]^

Visible‐light‐responsive monoclinic scheelite BiVO_4_ is emerging as a promising candidate photocatalyst due to its low cost, nontoxicity, high chemical stability, and suitable bandgap (2.4 eV).^[^ ^8^ ^]^ Nevertheless, the low specific surface area, non‐efficient charge separation, poor electron mobility, and short hole diffusion length limit the overall photocatalytic efficiency of bulk BiVO_4_.^[^ ^9^ ^]^ Although challenging, various strategies have been applied to overcome the inherent drawbacks of bulk BiVO_4_, improving its photocatalytic performance, such as doping,^[^ ^10^ ^]^ morphology and structure tailoring,^[^ ^11^ ^]^ and heterojunction construction.^[^ ^12^ ^]^ For example, in our previous work, we prepared novel visible‐light‐driven BiVO_4_@TiO_2_ microspheres with unique core@porous‐shell structures.^[^ ^6^ ^]^ Benefiting from the formed heterojunction between the BiVO_4_ and TiO_2_, and the intimate interface in the heterojunction as well as the unique porous‐shell structure, the well‐designed microspheres showed satisfactory photocatalytic elimination efficiency for both organic dyes and colorless organic contaminant. In recent years, 2D materials have attracted much attention for photocatalysis due to their large specific surface area, effective charge carrier separation and transport, and numerous surface sites for reactions.^[^ ^11^, ^13^ ^]^ The synthesis of 2D BiVO_4_ NSs has thus been explored with the hope of mitigating the above‐mentioned intrinsic issues of bulk BiVO_4_.^[^ ^14^ ^]^ Nevertheless, it has since been noted that although certain improvements can be obtained by tailoring BiVO_4_ into a 2D structure, the charge separation and transfer efficiency are still not effective enough for practical applications.

A well‐designed heterojunction can not only enhance the light absorption capability but also effectively promote the interfacial transfer of charge carriers, which are critical for the enhancement of photocatalytic performance.^[^ ^15^ ^]^ Engineering heterojunction by coupling BiVO_4_ with a suitable semiconductor is thus a promising way to further improve the overall photocatalytic activity. As an inexpensive and non‐toxic semiconductor photocatalyst, p‐type Cu_2_O with a narrow bandgap (≈2.2 eV) and large light absorption coefficient is a good candidate to construct heterojunctions with n‐type BiVO_4_.^[^ ^16^ ^]^ The constructed BiVO_4_/Cu_2_O p‐n heterojunction can efficiently promote the separation and interfacial transfer of charge carriers.^[^ ^17^ ^]^ In addition, as reported, the BiVO_4_/Cu_2_O composite also showed increased light absorption capacity than bare BiVO_4_, further enhancing the photocatalytic activity.^[^ ^18^ ^]^ However, such a type II heterojunction inevitably reduces the redox capacities of the entire system to some extent.^[^ ^19^, ^20^ ^]^ Specifically, in the Type II junction, the photo‐excited electrons transfer downward from the conduction band (CB) of semiconductor A to semiconductor B with a less negative CB edge; holes transfer upward from the valence band (VB) of semiconductor B to semiconductor A with less positive VB level. The final outcome is the decrease of the overall redox level of the entire system.^[^ ^21^ ^]^ To retain the initially strong redox capacity of respective components, Z‐scheme heterojunction was thus explored.^[^ ^22^, ^23^ ^]^ Similarly, a typical Z‐scheme heterojunction consists of semiconductor A with a higher CB position and semiconductor B with a lower VB position.^[^ ^24^, ^25^ ^]^ But in this case, the photo‐excited electrons in semiconductor B recombine with the photo‐generated holes in semiconductor A at the interface.^[^ ^26^ ^]^ Consequently, the remained electrons and holes accumulate in semiconductors A and B, respectively. As such, the strong reduction capability of semiconductor A and the strong oxidation capability of semiconductor B are maintained meanwhile, maintaining the overall high redox capacity of the entire system.^[^ ^27^ ^]^ To this end, Qin's group explored a novel Z‐scheme BiVO_4_/g‐C_3_N_4_ heterojunction for removing organic dyes. Benefiting from the Z‐scheme structure, the BiVO_4_/g‐C_3_N_4_ composite showed significantly enhanced photocatalytic activity for degrading rhodamine B, which was ≈12.5 and 2.4 times higher than that of the pure BiVO_4_ and g‐C_3_N_4_, respectively.^[^ ^28^ ^]^ To construct the BiVO_4_/Cu_2_O Z‐scheme heterojunction, an electron mediator, such as metallic nanoparticles (NPs), is usually required in the junction, which can efficiently promote the interfacial charge transfer.^[^ ^29^ ^]^ Further considering the intriguing localized surface plasmon resonance (LSPR) effect of certain metal NPs in the visible range,^[^ ^30^, ^31^ ^]^ it is wise to choose the electron mediator that can play more roles in visible photocatalysis.

Herein, we prepared ultrathin BiVO_4_ NSs with several nanometers in thickness, which provided more reaction sites and reduced the charge carrier transfer length to the reaction sites at first. A novel BiVO_4_‐Au‐Cu_2_O composite was subsequently well designed, in which by creating the Z‐scheme and type II heterojunctions multiple charge transfer paths were enabled on the ultrathin BiVO_4_ NSs surfaces. Compared to the conventional type II BiVO_4_/Cu_2_O heterojunction, the BiVO_4_‐Au‐Cu_2_O composite showed significantly enhanced photocatalytic activity in the degradation of tetracycline (Tc) under visible light irradiation. This was attributed to the synergism of the ultrathin 2D structure of BiVO_4_ NSs, the LSPR effect of the Au NPs, multiple charge transfer paths, and strong redox capacity of the photo‐excited charge carriers. This study can provide a guide for the development of efficient visible‐light‐driven BiVO_4_‐based photocatalysts for the removal of persistent organic contaminants, such as antibiotics.

## 2 Results and Discussion

### 2.1 Preparation and Characterization of the Nanosheets

**Figure 1:**
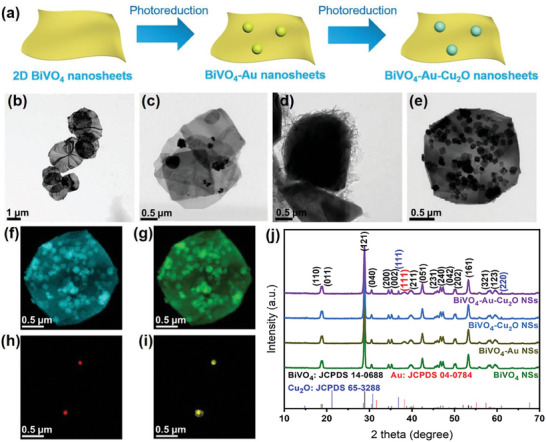
a) Scheme for the preparation of the BiVO_4_‐Au‐Cu_2_O NSs; TEM images of the b) BiVO_4_ NSs, c) BiVO_4_‐Au NSs, d) BiVO_4_‐Cu_2_O NSs, and e) BiVO_4_‐Au‐Cu_2_O NSs; f–i) EDS mapping images for the element of Bi, V, Au, and Cu in the BiVO_4_‐Au‐Cu_2_O NSs, respectively; j) XRD spectra of the BiVO_4_ NSs and nanohybrids. The standard XRD profiles of monoclinic scheelite BiVO_4_ (Black), Fcc Au (red), and cubic Cu_2_O (blue) are also included.

In this study, the BiVO_4_‐Au‐Cu_2_O NSs were synthesized starting from the ultrathin 2D BiVO_4_ NSs (**Figure** **1**a). Briefly, the 2D BiVO_4_ NSs were first synthesized, followed by the deposition of the Au NPs on the 2D BiVO_4_ NSs surface. The BiVO_4_‐Au‐Cu_2_O NSs were subsequently synthesized by selectively depositing Cu_2_O layers on the Au NPs through photoreduction. As a control sample, BiVO_4_‐Cu_2_O NSs were also prepared by directly depositing Cu_2_O on the BiVO_4_ NSs. TEM images (Figure S2, Supporting Information) confirmed the successful synthesis of the ultrathin 2D BiVO_4_ NSs. The apparent Moiré patterns were observed in the stacked areas, indicating the ultrathin structure of the BiVO_4_ NSs.^[^ ^32^ ^]^ Based on the AFM height profiles along the gray, red and blue lines (Figure S3, Supporting Information), the thickness of three observed BiVO_4_ NSs were estimated to be 4.3, 5.3, and 5.4 nm, respectively. EDS mapping images confirmed the uniform distribution of Bi, V, and O elements in the BiVO_4_ NS (Figure S4, Supporting Information). After ligand change, the morphology of the BiVO_4_ NSs remained unchanged (Figure 1b). In the TEM image of the BiVO_4_‐Au NSs, higher contrast NPs were observed on the BiVO_4_ NSs surface (Figure 1c), which were supposed to be the deposited Au NPs. This was supported by EDS mapping images, where aggregated Au signal was shown in certain areas with respect to basically uniform distribution of Bi, V, and O (Figure S5, Supporting Information). Cu_2_O was deposited following the similar photoreduction procedure with a fluffy structure observed around the BiVO_4_ NSs (Figure 1d). EDS mappings confirmed the presence of Cu_2_O layers on the BiVO_4_ NSs surface (Figure S6, Supporting Information). For the BiVO_4_‐Au‐Cu_2_O NSs, some small sheets and NPs observed on the BiVO_4_ NSs should be BiVO_4_ fragments broken by the high‐power ultrasonication (Figure 1e). EDS mappings revealed aggregated Au NPs in specific areas of the BiVO_4_ NSs, with Cu accumulation at the same locations, suggesting selective assembly of Cu_2_O onto Au NPs during the photoreduction process (Figure 1f–i). The formation of the BiVO_4_/Au/Cu_2_O favors the formation of Z‐scheme heterojunction as Au NPs can serve as an electron mediator to facilitate the charge transfer between the Cu_2_O and BiVO_4_.

XRD analysis was conducted to analyze the crystal structures of the photocatalysts (Figure 1j). The standard cards of monoclinic scheelite BiVO_4_ (JCPDS No. 14–0688), face‐centered cubic (Fcc) Au (JCPDS card No. 04–0784), and cubic Cu_2_O (JCPDS card No. 65–3288) are also shown as references. The XRD pattern of BiVO_4_ NSs showed peaks at the 2θ of 18.8°, 19.0°, 28.8°, 30.6°, 34.6°, 34.8°, 39.7°, 42.5°, 47.0°, 47.3°, 50.3, 55.3°, 59.1°, and 59.6°, which are well indexed to the (110), (011), (121), (040), (200), (002), (211), (051), (231), (240), (042), (202), (161), (321), and (123) planes of monoclinic scheelite BiVO_4_.^[^ ^33^, ^34^, ^35^ ^]^ The BiVO_4_‐Au NSs showed an additional peak at 38.8°, corresponding to the (111) plane of Fcc Au; the BiVO_4_‐Cu_2_O NSs displayed additional peaks at 36.9° and 61.3°, assigned to the (111) and (220) planes of cubic Cu_2_O, respectively. As for the BiVO_4_‐Au‐Cu_2_O NSs, characteristic peaks of monoclinic scheelite BiVO_4_, Fcc Au, and cubic Cu_2_O were all detected, confirming the successful preparation of the ternary composite.

### 2.2 X‐ray Photoelectron Spectroscopy (XPS) Analysis

XPS spectra were acquired to analyze surface chemical composition and valence states of the photocatalysts (Figures S7–S10, Supporting Information). The XPS spectra of the BiVO_4_ NSs showed the characteristics of Bi, V, and O (Figure S7, Supporting Information).^[^ ^36^ ^]^ For the XPS spectra of the BiVO_4_‐Au NSs (Figure S8, Supporting Information), additional peaks observed at ≈83.8 and 87.6 eV were attributed to Au 4f_7/2_ and Au 4f_5/2_, respectively, suggesting the successful deposition of Au NPs on the BiVO_4_ NSs surface.^[^ ^37^ ^]^ The XPS spectra of the BiVO_4_‐Cu_2_O NSs show peaks at ≈932.6 and 952.3 eV, which were assigned to Cu 2p_3/2_ and Cu 2p_3/2_ of Cu^+^ (Figure S9, Supporting Information), revealing the formation of the Cu_2_O.^[^ ^38^ ^]^ All the above‐mentioned characteristic peaks of Bi, V, O, Au, and Cu could be identified in the XPS spectra of the BiVO_4_‐Au‐Cu_2_O NSs (Figure S10, Supporting Information), confirming the successful deposition of both Au NPs and Cu_2_O layers onto the BiVO_4_ NSs.

**Figure 2:**
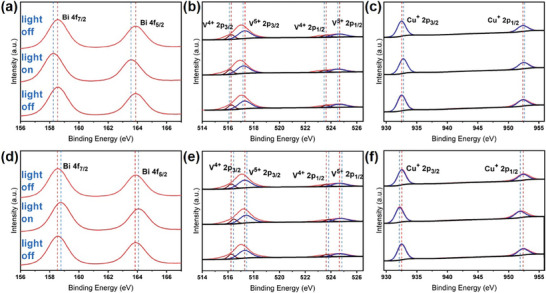
High‐resolution XPS spectra of the element of a) Bi, b) V, and c) Cu in the BiVO_4_‐Cu_2_O NSs during a light off‐on‐off cycle; high‐resolution XPS spectra of the element of d) Bi, e) V, and f) Cu in the BiVO_4_‐Au‐Cu_2_O NSs during a light off‐on‐off cycle. The 365 nm illumination light was generated by an LED lamp.

Previous study has revealed that the transfer of the photo‐generated charge carrier in the heterojunction depends on the structure of the heterojunction. The reversible binding energy shifts of the involved components under illumination can reflect light‐induced charge transfer and confirm the structure of the heterojunction.^[^ ^39^ ^]^ To this end, the XPS measurements were performed for the BiVO_4_‐Cu_2_O NSs and BiVO_4_‐Au‐Cu_2_O NSs during a light (365 nm generated by an LED light) off‐on‐off cycle to probe charge transfer between different components (**Figure** **2**). Under 365‐nm light illumination, compared with the pristine BiVO_4_ and Cu_2_O, the BiVO_4_‐Cu_2_O NSs showed decreased binding energies of 0.2 eV for Bi and V, and an increased binding energy of 0.2 eV for Cu (Figure 2a–c). When the light was turned off, the binding energies returned to their initial values, excluding the possibility of photo‐induced material oxidation/reduction. It also revealed the photo‐generated electron transfer from the Cu_2_O to BiVO_4,_ which was in line with the type‐II heterojunction. In clear contrast to the BiVO_4_‐Cu_2_O NSs, when the BiVO_4_‐Au‐Cu_2_O NSs were irradiated, the binding energies of Bi and V increased by 0.2 eV while that of Cu decreased by 0.2 eV (Figure 2d–f), indicating the formation of dominant Z‐scheme heterojunction in the ternary composite.^[^ ^39^, ^40^ ^]^ The binding energy shifts of the involved elements in both the BiVO_4_‐Cu_2_O NSs and BiVO_4_‐Au‐Cu_2_O were attributed to the chemical bond formed at the interface, which could promote the effective electron transfer between the different components.^[^ ^41^ ^]^ In the BiVO_4_‐Au‐Cu_2_O NSs, the Z‐scheme heterojunction formed between the BiVO_4_ and Cu_2_O, with the Au NPs acting as the efficient electron mediator. We would like to point out that this observation did not absolutely exclude the formation of the Type‐II heterojunction between the BiVO_4_ and Cu_2_O in this sample, since they did have certain, although limited, direct contact.

### 2.3 Optical Properties and Photoelectrochemical Measurements

**Figure 3:**
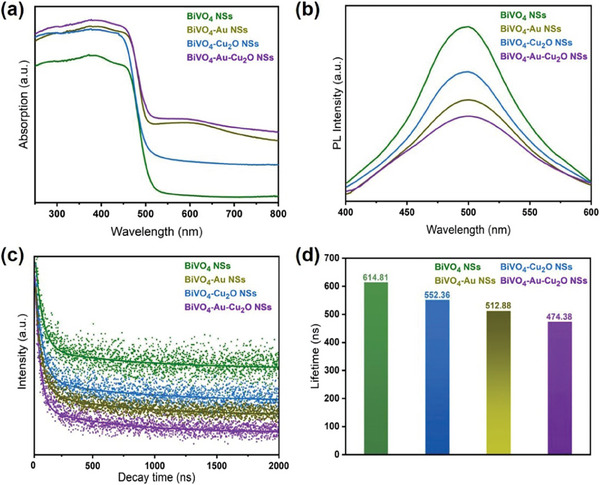
a) UV–vis absorption spectra; b) steady‐state PL spectra; c) time‐resolved PL spectra; d) lifetime of the BiVO_4_ NSs, BiVO_4_‐Au NSs, BiVO_4_‐Cu_2_O NSs, and BiVO_4_‐Au‐Cu_2_O NSs, respectively.

The optical properties of the as‐prepared samples can be straightforwardly seen by eyes since there were dramatic color changes among them (Figure S11, Supporting Information). The BiVO_4_ NSs and BiVO_4_‐Cu_2_O NSs appeared yellow, while the BiVO_4_‐Au NSs and BiVO_4_‐Au‐Cu_2_O NSs were green. UV–visible diffuse reflectance (DRS) spectra were measured to assess their optical properties quantitatively. As shown in **Figure** **3**a, the pure BiVO_4_ NSs showed light absorption ranging from UV to short‐wavelength visible light region with an absorption edge of 515 nm, corresponding to the bandgap of 2.4 eV.^[^ ^42^ ^]^ Compared to the pure BiVO_4_ NSs, all the BiVO_4_‐based nanohybrids showed enhanced UV and visible light absorption, which was attributed to the deposited Au and/or Cu_2_O. Different from the BiVO_4_ NSs and BiVO_4_‐Cu_2_O NSs, the BiVO_4_‐Au NSs and BiVO_4_‐Au‐Cu_2_O NSs exhibited additional broad absorption bands centering ≈590 nm, corresponding to the LSPR effect of Au NPs.^[^ ^43^ ^]^ It should be noticed that the LSPR peaks of Au NPs for both BiVO_4_‐Au NSs and BiVO_4_‐Au‐Cu_2_O NSs showed redshifts with respect to commonly reported Au LSPR peak at ≈520 nm.^[^ ^44^ ^]^ It is well known that the LSPR peak of Au NPs is sensitive to their size, shape, and immediate environments, including stabilizers and substrates.^[^ ^45^, ^46^ ^]^ In this study, the redshift of the LSPR peak to ≈600 nm should be attributed to both the relatively large size of the Au NPs and the presence of BiVO_4_ NSs as the substrate with a relatively high refractive index, which is consistent with previous studies.^[^ ^47^, ^48^ ^]^ The light absorption capacity of BiVO_4_‐Au‐Cu_2_O NSs was significantly enhanced compared to the bare BiVO_4_ NSs, which is beneficial for photocatalytic performance.

The steady‐state photoluminescence (PL) spectra of the photocatalysts were taken to investigate the photo‐generated charge carrier behavior in the heterojunction. The spectra were recorded in the range of 400 to 600 nm with the excitation wavelength of 375 nm. As shown in Figure 3b, all the photocatalysts exhibited PL emission peaks at ≈500 nm, corresponding to the radiative recombination of the photoinduced electrons and holes in BiVO_4_. As compared to the plain BiVO_4_ NSs, all the BiVO_4_‐based nanohybrids showed decreased peak intensities, suggesting reduced electron‐hole recombination. The BiVO_4_‐Au‐Cu_2_O NSs showed the lowest PL peak intensity, suggesting that the multiple charge transfer paths significantly facilitated the charge separation. On the contrast, only a single charge transfer path was expected in the BiVO_4_‐Cu_2_O NSs and BiVO_4_‐Au NSs.

The charge carrier transfer dynamics were further investigated by time‐resolved PL (TRPL) measurements (Figure 3c,d) and the derived lifetime components are shown in Table S1 (Supporting Information). The average lifetime for the BiVO_4_ NSs was calculated to be 614.81 ns, which is consistent with previous reports.^[^ ^49^, ^50^ ^]^ Compared to the pure BiVO_4_ NSs, the BiVO_4_‐Au NSs, BiVO_4_‐Cu_2_O NSs, and BiVO_4_‐Au‐Cu_2_O NSs showed decreased lifetimes of 512.88, 552.36, and 474.38 ns, respectively (Figure 3d). It suggested that the formed heterojunction on the BiVO_4_ NSs surface could promote the charge transfer in the BiVO_4_‐based photocatalysts. The BiVO_4_‐Au‐Cu_2_O NSs showed the shortest average lifetime, indicating the most efficient separation of the charge carriers, which is in agreement with the steady‐state PL result.

### 2.4 Photocatalytic Performance and Trapping Experiments

**Figure 4:**
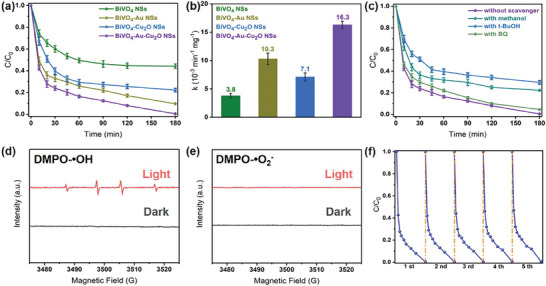
a) The plot of (C/C_0_) versus reaction time of Tc (30 mg L^−1^) under λ > 420 nm light irradiation in the presence of the photocatalysts; b) degradation rate constants of Tc (30 mg L^−1^) under λ > 420 nm light irradiation in the presence of the photocatalysts; c) photocatalytic degradation of Tc (30 mg L^−1^) without and in the presence of different scavengers under λ > 420 nm light irradiation; DMPO spin‐trapping EPR spectra of d) •OH and e) •O_2_^−^ radicals for BiVO_4_‐Au‐Cu_2_O NSs in the dark and under λ > 420 nm irradiation; f) photocatalytic stability of the BiVO_4_‐Au‐Cu_2_O NSs in Tc solution (30 mg L^−1^) in five successive cycles under λ > 420 nm. The duration of light illumination in each cycle is 3 h. The photocatalytic degradation of Tc in the presence of the photocatalysts and the trapping experiment by the BiVO_4_‐Au‐Cu_2_O NSs without and with the presence of different scavengers were carried out for three times, and both the average values and error bars are presented.

The photocatalytic activities of the as‐prepared samples were investigated by monitoring the degradation of Tc in aqueous solution. As shown in **Figure** **4**a, all the BiVO_4_‐based nanohybrid photocatalysts showed enhanced photocatalytic activities than the pure BiVO_4_ NSs under visible illumination (λ > 420 nm). The improved photocatalytic activity of the BiVO_4_‐Cu_2_O NSs was attributed to the formed type II heterojunction, which facilitated the charge transfer between the BiVO_4_ and Cu_2_O. The BiVO_4_‐Au NSs also exhibited higher Tc removal efficiency than the pure BiVO_4_ NSs, which was ascribed to the LSPR effect of Au NPs.^[^ ^51^ ^]^ The LSPR‐induced hot electrons with sufficient energy can overcome the Schottky barrier at metal/semiconductor interfaces, taking part in photocatalytic reaction and enhancing the photocatalysis efficiency.^[^ ^52^ ^]^ The highest photocatalytic activity was achieved by the BiVO_4_‐Au‐Cu_2_O NSs, in which case 80% of the initial Tc was removed after a short 30‐min visible light illumination. The reaction rate constants (*k*) were then calculated by fitting the degradation data with the first‐order reaction model (Figure 4b; Figure S12, Supporting Information) and normalized by the mass of added photocatalysts. The normalized *k* value of the BiVO_4_‐Au‐Cu_2_O NSs (16.3 × 10^−3^ min^−1^ mg^−1^) was ≈4.3, 1.6, and 2.3 times higher than that of the pure BiVO_4_ NSs (3.8 × 10^−3^ min^−1^ mg^−1^), BiVO_4_‐Au NSs (10.3 × 10^−3^ min^−1^ mg^−1^), and BiVO_4_‐Cu_2_O NSs (7.1 × 10^−3^ min^−1^ mg^−1^), respectively. The synergistic effect of the following unique features contributed to the enhanced photocatalytic activity of the BiVO_4_‐Au‐Cu_2_O NSs. The ultrathin 2D structure of the BiVO_4_ NSs provided abundant surface and reactive sites for the photocatalytic reaction. The formed multiple transfer paths on BiVO_4_ NSs surface effectively promoted the charge carrier separation and transfer, enhancing the photocatalytic performance. Specifically, the formed Z‐scheme heterojunction can not only efficiently suppress the charge carrier recombination but also preserve the strong redox capacity of the charge carriers for the Tc degradation. The presence of direct contact between the BiVO_4_ and Cu_2_O in some areas also allowed the formation of the type‐II heterojunction, providing an additional charge transport path. In addition, the deposited Au NPs increased the light‐harvesting capability due to the LSPR effect.^[^ ^53^, ^54^ ^]^

For better understanding the underlying mechanism of photocatalytic degradation of Tc in the presence of the BiVO_4_‐Au‐Cu_2_O NSs, which showed the highest photocatalytic activity among all the as‐prepared photocatalysts, trapping experiments were carried out to detect the involved active species in the presence of this photocatalyst by adding three different scavengers. As shown in Figure 4c, the photocatalytic activity only showed a slight decrease with the addition of BQ (a scavenger for •O_2_^−^). Significant decreases occurred with the addition of methanol (a hole scavenger) and t‐BuOH (a scavenger for •OH). It could be concluded that the •OH and h^+^ played more important roles in the degradation of Tc under visible light irradiation, while the contribution of •O_2_^−^ was negligible.

To further detect the involved reactive species during the photocatalysis process, the EPR spectra were acquired. It can be seen that no EPR peaks were detected in the presence of the BiVO_4_‐Au‐Cu_2_O NSs in the dark (Figure 4d). While under light illumination, four peaks with the intensity ratio of 1:2:2:1 could be observed at the g value of 2.006, which were attributed to the DMPO‐•OH.^[^ ^55^ ^]^ The generation of the characteristic peaks of DMPO‐•OH revealed that the hole and hole induced •OH were the reactive species involved in the photocatalytic reaction. Different from the DMPO‐•OH, no DMPO‐•O_2_^−^ peak was conducted both in the dark and under light illumination (Figure 4e), revealing that the contribution of •O_2_^−^ to the photocatalytic reaction was negligible, which was consistent with the results of the trapping experiments.

The stability of a photocatalyst is critical for its practical application in terms of the economic cost and recovery capacities. In order to investigate the stability of the BiVO_4_‐Au‐Cu_2_O NSs, which showed the best photocatalytic performance among the as‐prepared photocatalysts, the photocatalytic degradation activities for Tc were measured in five successive cycles under λ > 420 nm illumination (Figure 4f). The plots of ‐ln(C/C_0_) versus reaction time of Tc solution in the presence of the BiVO_4_‐Au‐Cu_2_O NSs are drawn and the corresponding degradation rate constants in the presence of the BiVO_4_‐Au‐Cu_2_O NSs were subsequently calculated (Figure S13, Supporting Information). They were 16.3, 16.1, 15.8, 15.6, and 15.4 (×10^−3^ min^−1^ mg^−1^) in the five successive cycles, respectively. So ≈95.6% of the original photocatalytic activity was remained for the BiVO_4_‐Au‐Cu_2_O NSs after five successive cycles, suggesting the excellent stability of the BiVO_4_‐Au‐Cu_2_O NSs, which is beneficial to the practical application of photocatalysis.

### 2.5 Possible Degradation Pathway of Tc

**Figure 5:**
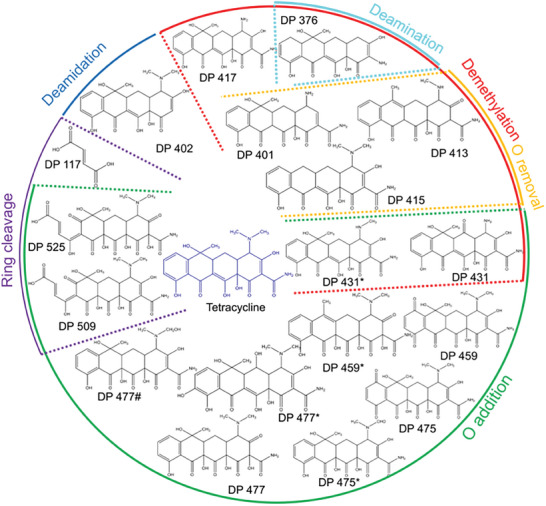
Possible degradation pathways of T_C_ in the presence of the BiVO_4_‐Au‐Cu_2_O NSs.

The degradation of antibiotics by photocatalysis may generate not‐fully degraded products, which might be even more toxic than their parent products.^[^ ^56^ ^]^ Therefore, the identification of the by‐products produced during photocatalysis and the study of their toxicity is of great importance. Figures S14 and S15 (Supporting Information) showed the concentrations of the degradation products (DPs), detected by an ultra‐high performance liquid chromatography‐mass spectrometer (UHPLC‐MS), in the solutions treated by the BiVO_4_‐Au‐Cu_2_O photocatalyst for different time. The concentrations of most of the DPs followed the same trend, first increased within 20–30 min and then decreased to a negligible level after 30 min or longer time. The only exception was DP 117, whose concentration kept increasing during the reaction period up to 120 min. So most of these DPs acted as relatively short‐living intermediates during degradation paths.^[^ ^57^, ^58^ ^]^ For instance, the deamidation of Tc led to the formation of DP 402, while the demethylation of Tc first led to by‐products such as DP 417, and then DP 376 by deamination.^[^ ^59^, ^60^ ^]^ Demethylation and oxygen addition to the phenolic ring were thus the main degradation pathways of Tc in this study. This conclusion is in line with that the methyl groups of Tc were identified as the “easy” sites to attack by density functional theory and Fukui index.^[^ ^61^ ^]^ It is worth mentioning that the exact Tc degradation processes are difficult to be completely unveiled because of the detection of multiple chemicals simultaneously and the lack of time resolution of the analytical technique. Even so, based on the detected intermediates, Tc degradation in this study could be mainly categorized into demethylation, deamination, deamidation, oxygen addition, oxygen removal, and ring cleavage (**Figure** **5**).

### 2.6 Antibacterial Activity of the Tc Degradation Products

**Figure 6:**
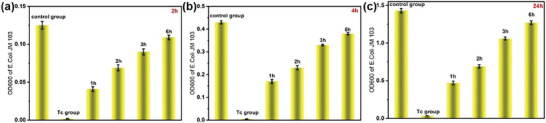
OD_600_ of E. coli incubated for a) 2, b) 4, and c) 24 h in the control group, untreated Tc solution and photocatalytic effluents after different photodegradation times (1, 2, 3, and 6 h).

Although the photocatalysis results revealed that Tc was eliminated effectively in the presence of the photocatalysts, the generated by‐products may show toxicity even at trace concentrations.^[^ ^62^ ^]^ The photocatalytic detoxification efficiency of Tc solution by the BiVO_4_‐Au‐Cu_2_O NSs was assessed by using E coli as a model microorganism. As shown in **Figure** **6**, high optical densities of the *E. coli* could be observed for the control group incubated for both 2, 4, and 24 h, indicating the suitability of the mixture of LB media and DI water for bacteria growth. In the Tc solution (30 mg L^−1^) before the photocatalytic treatment, the optical density remained very low even though the bacteria were incubated for 24 h, indicating that the bacterial growth was significantly inhibited by the biotoxicity of Tc. The microorganism growth, however, increased gradually with elongating photocatalytic treatment time of Tc solutions from 1 to 6 h, suggesting the toxicity of the treated Tc solution to biospecies decreased. In particular, the microorganism growth in the 6 h‐treated Tc solution was similar to that in the control group, demonstrating the high photocatalytic detoxification efficiency of the BiVO_4_‐Au‐Cu_2_O NSs. But even low, the small toxicity did remain, which may be attributed to the recalcitrant by‐products that survived from the photocatalytic treatment. Accordingly, the Ecological Structure Activity Relationship (*ECOSAR*) program was employed to determine the level of toxicity of the detected DPs in this study.^[^ ^63^, ^64^ ^]^ As shown in **Table** **1**, certain DPs, such as DP 401, DP 413, DP 459^*^, and DP 477, are categorized as “very toxic” by the *ECOSAR* program based on the functional groups of their chemical structure. These DPs might be toxic against microorganisms and inhibit their growth to some extent.^[^ ^65^ ^]^

**Table 1:** Toxicity prediction of the identified DPs in treated Tc solutions by using the ECOSAR program.

DPs	Acute Toxicity [mg L^−1^]			Chronic Toxicity (ChV) [mg L^−1^]		
	Fish (96 h) LC_50_	Daphnid (48 h) LC_50_	Green Algae (96 h) EC_50_	Fish	Daphnid	Green Algae
DP 117	53 300	25 300	8960	4220	1500	1570
DP 376	88.1	356	18.3	579	87.3	170
DP 401	24	1.82	0.924	0.216	0.381	0.635
DP 402	37.2	3.09	5.24	0.544	0.693	1.48
DP 413	5.66	0.961	1.19	0.070	0.186	0.227
DP 415	27.7	2.56	3.40	0.383	0.558	1.07
DP 417	90.6	5.72	18.3	1.54	1.38	3.84
DP 431^*^	68.5	4.78	12.1	1.10	1.12	2.84
DP 431	183	9.31	49.0	3.51	2.37	8.19
DP 459	1420	402	126	58.0	24.6	132
DP 459^*^	1.01	0.520	0.107	0.022	0.092	0.080
DP 475	606	194	63.7	28.6	12.6	72.0
DP 475^*^	56.9	4.35	2.57	0.871	0.997	2.31
DP 477	6.26	1.09	1.33	0.078	0.21	0.254
DP 477^*^	254	12.0	74.9	5.07	3.12	11.6
DP 477^#^	154	4.63	17.7	0.967	1.07	2.54
DP 509	388	1290	19.0	97.4	24.1	5.00
DP 529	98.6	117	7.09	27.5	13.4	48.5

Very toxic (red color): LC50/EC50/ChV<1 mg L^−1^; toxic (yellow color): 1 mg L^−1^ <LC50/EC50/ChV<10 mg L^−1^; harmful (lilac color): 10 mg L^−1^<LC50/EC50/ChV<100 mg L^−1^, not harmful (green color) LC50/EC50/ChV>100 mg L^−1^. LC50 refers to the amount of a substance suspended in the air required to kill 50% of the test animals during a predetermined observation period. EC50 refers to half maximal effective concentration. ChV is defined as the geometric mean of the no‐observed‐effect concentration and the lowest observed effect concentration.

### 2.7 Mechanism During the Photocatalysis

**Figure 7:**
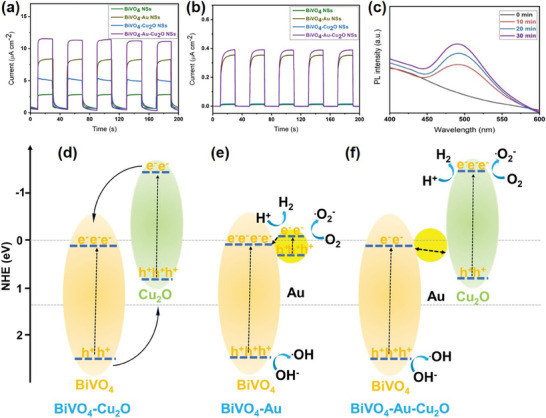
Photocurrent response during the light on‐and‐off cycles of the BiVO_4_ NSs, BiVO_4_‐Au NSs, BiVO_4_‐Cu_2_O NSs, and BiVO_4_‐Au‐Cu_2_O NSs a) without and b) with a long‐pass filter (530 nm), respectively; c) PL spectral change of the coumarin solution in the presence of the BiVO_4_‐Au‐Cu_2_O NSs under visible light illumination; the scheme of the photocatalytic mechanism of the d) BiVO_4_‐Cu_2_O NSs, e) BiVO_4_‐Au NSs, and f) BiVO_4_‐Au‐Cu_2_O NSs during the photocatalysis process.

The PEC tests were conducted to investigate the contribution of the multiple charge transfer paths formed on the BiVO_4_ NSs surface and the LSPR effect of the Au NPs (**Figure** **7**a,b; Figure S16, Supporting Information). Figure 7a shows the transient photocurrents during the light on/off cycles. For all the photocatalysts, there was almost no current in the dark and the current density significantly increased under simulated solar light illumination. All the nanohybrid photocatalysts showed higher photocurrent densities than the BiVO_4_ NSs, which confirmed that the formed heterojunction could effectively promote the charge carrier separation and transfer.^[^ ^66^ ^]^ Notably, the BiVO_4_‐Au‐Cu_2_O NSs exhibited the highest photocurrent density (11.7 µA cm^−2^), which was 4.3, 1.3, and 2.3 times that of the BiVO_4_ NSs (2.7 µA cm^−2^), BiVO_4_‐Au NSs (8.2 µA cm^−2^), and BiVO_4_‐Cu_2_O NSs (5.1 µA cm^−2^), respectively. The significantly increased photocurrent density for the BiVO_4_‐Au‐Cu_2_O NSs should be ascribed to the formed multiple charge transfer paths and the LSPR effect of the Au NPs. With the presence of multiple charge transfer paths, charge transfer became more effective,^[^ ^67^ ^]^ as also supported by the smallest arc radius in the electrochemical impedance spectroscopy (EIS) Nyquist plot of the BiVO_4_‐Au‐Cu_2_O NSs (Figure S16, Supporting Information). To verify the contribution of the LSPR effect of Au NPs to photocatalysis, the transient photocurrent was also measured under λ > 530 nm light illumination (Figure 7b). BiVO_4_ NSs and BiVO_4_‐Cu_2_O NSs showed almost no current, while obvious photocurrents were detected for BiVO_4_‐Au NSs and BiVO_4_‐Au‐Cu_2_O NSs, confirming that the LSPR effect of Au NPs extended the photo‐response range.

As stated above, the XPS analysis measured during a light off‐on‐off cycle verified that the charge transfer in the BiVO_4_‐Au‐Cu_2_O NSs mainly followed the Z‐scheme direction. In order to double confirm it in the BiVO_4_‐Au‐Cu_2_O NSs, the redox capabilities of the composite were further tested. Previous study found that coumarin can be transformed to 7‐hydroxylcoumarin in the presence of •OH, which is mainly produced from the oxidation of OH^−^ by photo‐generated holes.^[^ ^68^ ^]^ The formed 7‐hydroxylcoumarin can be easily detected since it shows the PL emission peak at 490 nm with the excitation at 325 nm.^[^ ^69^ ^]^ If the traditional type II heterojunction was the only junction formed in the BiVO_4_‐Au‐Cu_2_O NSs, the holes accumulated in the VB of the Cu_2_O would not be able to produce •OH because the VB position of Cu_2_O (0.87 eV vs RHE) is more negative than OH^−^/•OH oxidation potential (2.3 eV vs RHE).^[^ ^70^, ^71^ ^]^ In contrast, the formation of the Z‐scheme junction would allow the holes accumulated at the VB (2.4 eV vs RHE) of the BiVO_4_, which can oxidize OH^−^ to •OH that then reacts with coumarin to form 7‐hydroxylcoumarin, leading to the PL emission at 490 nm. As shown in Figure 7c, the coumarin solution, in the presence of the BiVO_4_‐Au‐Cu_2_O NSs, yielded the emission peak at 490 nm after being illuminated by visible light (λ ≥ 420 nm) for 10 min, and moreover, its intensity increased with increasing the irradiation time, suggesting the production of more •OH radicals with photocatalysis time.^[^ ^6^ ^]^ The production of •OH was another evidence of the presence of Z‐scheme heterojunction in the BiVO_4_‐Au‐Cu_2_O NSs. As a negative example, the BiVO_4_‐Cu_2_O NSs were also tested under the same conditions as comparison. For the BiVO_4_‐Cu_2_O NSs, under the light illumination, the photo‐generated electron transferred from the CB of Cu_2_O to the CB of BiVO_4_ (−0.16 eV vs RHE), while the photo‐reduced holes transferred from the VB of BiVO_4_ to the VB of Cu_2_O (0.87 eV vs RHE), which cannot oxidize OH^−^ to •OH.^[^ ^6^ ^]^ As a consequence, 7‐hydroxylcoumarin would not generate without the presence of •OH, and no PL emission peak could be detected. It can be seen that the BiVO_4_‐Cu_2_O NSs showed no PL emission peak for the 7‐hydroxylcoumarin (Figure S17, Supporting Information), suggesting that the heterojunction in the BiVO_4_‐Cu_2_O NSs was type II, while that in the BiVO_4_‐Au‐Cu_2_O NSs was Z‐scheme.

To further evaluate the formation of the Z‐scheme heterojunction, photocatalytic hydrogen evolution reaction (HER) was also carried out. The underlying logic is as follows. If only a type II heterojunction was formed, the photo‐generated electrons would transfer from the CB of the Cu_2_O to the CB of the BiVO_4_. As the CB position of BiVO_4_ (0.28 eV vs RHE) is more positive than the HER potential (0 eV vs RHE), the electrons would not be able to produce hydrogen.^[^ ^72^, ^73^, ^74^ ^]^ In contrast, in the Z‐scheme heterojunction, electrons would accumulate in the CB of the Cu_2_O, possessing a sufficiently negative energy level (−0.2 eV vs RHE) for producing hydrogen.^[^ ^75^ ^]^ This was exactly what we observed. Essentially the BiVO_4_‐Au‐Cu_2_O NSs clearly demonstrated photocatalytic HER activity (Figure S18, Supporting Information), while neither the BiVO_4_‐Cu_2_O NSs nor the BiVO_4_ NSs showed any activity in this reaction. The BiVO_4_‐Au NSs also led to H_2_ production, which was attributed to the LSPR‐induced hot electrons in the Au NPs.^[^ ^76^ ^]^ The LSPR‐excited hot electrons with high energy can not only be injected into the conduction band of the BiVO_4_ NSs but may also be partially involved in the photocatalytic reaction and the H_2_ was subsequently produced.^[^ ^77^ ^]^ It could also be seen that the BiVO_4_‐Au‐Cu_2_O NSs showed much better photocatalytic activity for H_2_ evolution than that of the BiVO_4_‐Au NSs. The significant difference of the photocatalytic HER activities among the different photocatalysts points to the formation of the Z‐scheme heterojunction in the BiVO_4_‐Au‐Cu_2_O NSs and the important contribution to the generation of H_2_.

Based on the above analysis, the photocatalytic mechanisms of the BiVO_4_‐Cu_2_O NSs, BiVO_4_‐Au NSs, and BiVO_4_‐Au‐Cu_2_O NSs were proposed (Figure 7d–f). For the BiVO_4_‐Cu_2_O NSs, the enhanced photocatalytic activity in the degradation of Tc was attributed to the formed type II heterojunction. The photo‐induced electrons transferred from the CB of the Cu_2_O to the CB of the BiVO_4_ while the holes were left in the VB of the Cu_2_O (Figure 7d). The electrons and holes were thus spatially separated, resulting in enhanced photocatalytic activity.^[^ ^78^ ^]^ The BiVO_4_‐Au NSs exhibited significantly improved photocatalytic performance than that of the pure BiVO_4_ NSs, which was contributed by the LSPR effect of Au NPs (Figure 7e). Benefiting from the LSPR effect of the Au NPs, the Au NPs enhanced the light absorption and the LSPR‐excited hot electrons may transfer to the CB of BiVO_4_ to promote the effective charge carrier separation, or may directly participate in the photocatalytic reaction, both of which increased the photocatalysis efficiency.^[^ ^79^ ^]^ In the BiVO_4_‐Au‐Cu_2_O NSs, the multiple charge transfer paths were constructed and effectively promoted the charge carrier separation and transfer, leading to superior photocatalytic performance in the degradation of Tc. Notably, the charge transfer mainly followed the Z‐scheme direction and perhaps partially followed the Type‐II direction where Cu_2_O and BiVO_4_ were directly interfaced (Figure 7f). To investigate the contributions of different components in the Z‐scheme heterojunction, the NAA test was performed. The NAA results revealed that the weight ratios for the BiVO_4_, Au and Cu_2_O in the BiVO_4_‐Au‐Cu_2_O NSs were 97.6%, 2.1%, and 0.3%, respectively. In the formed Z‐scheme heterojunction, the photo‐generated electrons in the CB of the BiVO_4_ mostly transfer to the interface and recombine with the holes from the VB of the Cu_2_O with the Au NPs acting as an effective conductor.^[^ ^80^ ^]^ As a result, the strong reduction and oxidation capacity were retained with electrons and holes accumulated in the CB of the Cu_2_O and the VB of the BiVO_4_, respectively.^[^ ^22^, ^23^ ^]^ The accumulated holes reacted with H_2_O or OH^−^ to generate •OH, while the electrons in the Cu_2_O reacted with O_2_ to produce •O_2_^−^ or participated in the HER. The Au NPs not only acted as the charge transfer conductor, but also extended the photo‐response range and generated hot electrons with sufficient energy via the LSPR effect.

## 3 Conclusion

In summary, ultrathin BiVO_4_ NSs with several nanometers in thickness were synthesized. The BiVO_4_‐Au NSs, BiVO_4_‐Cu_2_O NSs, and BiVO_4_‐Au‐Cu_2_O NSs were subsequently prepared by sequentially depositing Au and/or Cu_2_O on the BiVO_4_ NSs surface with a simple photoreduction method. Compared to the bare BiVO_4_ NSs, all the BiVO_4_ nanohybrids showed significantly enhanced photocatalytic activities. The BiVO_4_‐Au‐Cu_2_O NSs showed the best photocatalytic performance in the degradation of Tc due to the synergism of the 2D NSs structure, multiple charge transfer paths and the deposited Au NPs. The ultrathin 2D structure of the BiVO_4_ NSs provided sufficient surface area for the photocatalytic reaction. As an effective electron mediator, the Au NPs promoted the formation of the Z‐scheme heterojunction in the BiVO_4_‐Au‐Cu_2_O NSs. In addition, benefiting from the LSPR effect of the Au NPs, the light absorption of the photocatalysts was enhanced and the LSPR‐induced hot electrons may directly participate in the photocatalytic reaction. The multiple charge transfer paths effectively promoted the separation and transfer of the charge carrier. Specifically, the Z‐scheme heterojunction in the BiVO_4_‐Au‐Cu_2_O NSs, which was confirmed by XPS measurements during a light off‐on‐off cycle and the redox capability tests of the photo‐generated charge carriers, can not only facilitate the charge carrier separation and transfer but also maintain the original strong redox potentials of respective component in the heterojunction. As a consequence, a high Tc removal rate of 80% under 30 min visible light irradiation was achieved in the presence of the BiVO_4_‐Au‐Cu_2_O NSs. The trapping experiments and EPR measurement confirmed that the involved reactive species during the photocatalysis process were hole and hole‐induced •OH. The optical density method and ECOSAR program further confirmed the high efficiency of the BiVO_4_‐Au‐Cu_2_O NSs for the detoxification of Tc. The degradation products (DPs) during the photocatalysis were confirmed by using UHPLC and the plausible degradation pathways of Tc were consequently proposed. This work is expected to pave a strategy to prepare high‐performance visible‐light‐responsive photocatalysts with multiple charge transfer paths for antibiotic removal.

## 4 Experimental Section

### Materials

Bismuth nitrate pentahydrate (Bi(NO_3_)_3_·5H_2_O, 99.99%), ammonium metavanadate (NH_4_VO_3_, 99.9%), gold(III) chloride solution (HAuCl_4_·2H_2_O, 99.99%), copper(II) chloride dehydrate (CuCl_2_·2H_2_O, 99.95%), sodium dodecyl sulfate (SDS, 99.90%), hydroxylamine hydrochloride (NH_2_OH·HCl, 99.995%), benzoquinone (BQ), tertiary butanol (t‐BuOH), mercaptopropionic acid (MPA, 95%), octadecene (ODE, 90%), oleylamine (OLA, 70%), oleic acid (OA, 90%), and nitric acid (HNO_3_, 70%) were purchased from Sigma‐Aldrich Co. Ammonium hydroxide (NH_3_·H_2_O, 25%), hexane, methanol, ethanol, isopropyl alcohol (IPA), and nitric acid were purchased from Fisher Scientific Company. All of the reagents were used as received. The deionized (DI) water used during the study was obtained from a Millipore Ultrapure water system.

### Synthesis of BiVO_4_ NSs

The BiVO_4_ NSs were synthesized through a wet chemical method. First, 1 mmol of NH_4_VO_3_ was dissolved in 10 mL of water and 2 mL of HNO_3_. Meanwhile, 0.5 mmol of Bi(NO_3_)_3_·5H_2_O was added into the mixed solution of ODE (10 mL), OA (1 mL), and OLA (1 mL) in a 50 mL three‐neck flask. The mixture was then heated to 170 °C under N_2_ atmosphere until it became transparent. The solution was subsequently cooled down to 130 °C, and the as‐prepared NH_4_VO_3_ solution was injected. The reaction was further carried out at 100 °C for 40 min under N_2_ atmosphere. Afterward, it was quenched to room temperature in ice water quickly. After the addition of hexane/ethanol solution (30 mL/6 mL), the reaction solution was centrifuged to remove the unreacted precursors. The obtained product was washed with hexane/ethanol solution (30 mL/6 mL) for three times until the liquid supernatant became colorless. Finally, the product was dried at 80 °C overnight for use.

### Ligand Exchange of the BiVO_4_ NSs

For the practical application, the as‐synthesized BiVO_4_ NSs need to be transferred from hydrophobic to hydrophilic through ligand exchange. For the ligand exchange, 0.1 g of the as‐synthesized BiVO_4_ NSs was dispersed in 10 mL of hexane under magnetic stirring followed by adding the mixed solution of methanol (5 mL), MPA (180 µL) and ammonia solution (280 µL), and vigorously stirred for one more hour. Hexane/IPA solution (20 mL/5 mL) was then added, and the solution was centrifuged. Finally, the product was washed by deionized water and ethanol for three times, and dried in an oven at 80 °C overnight.

### Synthesis of BiVO_4_‐Au NSs

The BiVO_4_‐Au NS was prepared by depositing Au NPs onto the 2D BiVO_4_ NS surface through a photo‐assisted reduction reaction process. First, 0.1 g of the as‐prepared 2D BiVO_4_ NSs was dispersed in the mixture solution of DI water (100 mL) and methanol (1 mL) through sonication for 30 min. Afterward, different amounts of HAuCl_4_·2H_2_O solutions (0.056 m) were added, yielding a nominal Au loading level of 1 wt% (12 µL), 2 wt% (24 µL), 3 wt% (34 µL), 4 wt% (48 µL), and 5 wt% (60 µL) on the BiVO_4_ NSs. The photoreduction reaction of Au^3+^ ions to Au NPs was carried out under 3 h light illumination (300 W Xe lamp) with vigorous stirring. After the photoreduction process, the product was washed with DI water for three times and dried at 80 °C overnight. The as‐prepared BiVO_4_‐Au photocatalysts were named as BiVO_4_‐Au‐1, BiVO_4_‐Au‐2, BiVO_4_‐Au‐3, BiVO_4_‐Au‐4, and BiVO_4_‐Au‐5, whose photocatalytic degradation activities for Tc (30 mg L^−1^) were subsequently detected (Figure S1, Supporting Information).

### Synthesis of BiVO_4_‐Au‐Cu_2_O NSs

The BiVO_4_‐Au‐Cu_2_O NSs were synthesized from the as‐prepared BiVO_4_‐Au‐3 NSs through the photoreduction method similar to that for the Au NPs deposition, since the BiVO_4_‐Au‐3 NSs showed the best photocatalytic performance among the BiVO_4_‐Au NSs. In this study, the BiVO_4_‐Au‐3 NSs were denoted as BiVO_4_‐Au NSs for convenience. For the synthesis of the BiVO_4_‐Au‐Cu_2_O NSs, 0.05 g of the BiVO_4_‐Au NSs were dispersed in 100 mL of water followed by the addition of CuCl_2_ solution (3 mL, 0.1 M), methanol (1 mL), and SDS (2 g) and 1 h of vigorous stirring. Afterward, NH_2_OH solution (5 mL, 0.1 M) was dropwise added into the BiVO_4_‐Au suspension. The photoreduction reaction was carried out under light illumination of a 300 W Xe lamp for 4 h. The product was washed with DI water for three times and dried at 50 °C under vacuum.

### Synthesis of BiVO_4_‐Cu_2_O NSs

The BiVO_4_‐Cu_2_O NSs were also synthesized via the similar photoreduction method as the preparation of the BiVO_4_‐Au‐Cu_2_O NSs. Briefly, 0.1 g of the as‐prepared BiVO_4_ NSs were dispersed in 100 mL of water followed by the addition of CuCl_2_ solution (3 mL, 0.1 M), methanol (1 mL), and SDS (2 g) and vigorously stirred for 1 h. NH_2_OH solution (5 mL, 0.1 M) was then dropwise added, and the reaction was allowed to proceed for 4 h under continuous light illumination (300 W Xe lamp). The product was washed with DI water for three times and dried at 50 °C under vacuum.

### Characterization

Transmission electron microscopy (TEM) and energy dispersive X‐ray spectroscopy (EDX) were conducted on a JEOL 2100 F TEM with an acceleration voltage of 200 kV for morphology and composition analysis. X‐ray diffraction (XRD) patterns were recorded on a PANalytical X'Pert MRD X‐ray diffractometer equipped with a Cu Kα radiation (λ = 1.5406 Å). X‐ray photoelectron spectroscopy (XPS) was done on the VG Escalab 220I‐XL photoelectron spectrometer equipped with a twin anode Al Kα radiation as X‐ray source (hʋ = 1486.6 eV) to analyze the chemical composition of the samples. During the XPS test, the photocatalysts were detected in a light (365 nm, generated by an LED light) off‐on‐off operation cycle test. The weight ratios of the involved components for the sample were measured by the neutron activation analysis (NAA) method. The topography image of the pure ultrathin 2D BiVO_4_ NSs on a pre‐cleaned glass was detected by atomic force microscopy (AFM, Bruker, MultiMode 8) in a tapping mode. Optical absorption property was investigated through the UV–visible diffuse reflectance spectra (UV–vis DRS) recorded on a Varian Cary 5000 scan spectrometer equipped with an integrating sphere at room temperature. Steady‐state photoluminescence (PL) and time‐resolved PL (TRPL) spectra were obtained by a Horiba Jobin Yvon Fluorolog‐3 fluorescence spectrometer. Prior to the experiment, the photocatalyst powers were dispersed in water under ultrasonication. The absorption of the suspension of photocatalysts was detected by the UV–vis–NIR spectrometer to ensure that all the samples had similar absorption intensity at the excitation wavelength. Then the suspension was transferred into a quartz cuvette (1 cm × 1 cm × 3.5 cm) for the PL measurements. The steady‐state PL was recorded in the range of 400 to 600 nm with the excitation wavelength of 375 nm. The TRPL spectra were subsequently detected with the excitation of a pulsed laser with the wavelength of 408 nm before the decay. The average PL lifetime (τ) was estimated by fitting the TRPL decay curves following the equations:

(1) $$\mathrm{Fit}=A+B_{1}\exp\left\{{-}_{\tau_{1}}^{t}\right\}+B_{2}\exp\left\{{-}_{\tau_{2}}^{t}\right\}$$

(2) $$\tau =\frac{B_{1}\tau_{1}^{2}+B_{2}\tau_{2}^{2}}{B_{1}\tau_{1}+B_{2}\tau_{2}}$$

where A, B_1_, and B_2_ are constants obtained from the fitting of decay curves. τ_1_ and τ_2_ are different lifetime components resolved also from the fitting. The electron paramagnetic resonance (EPR) spectra were recorded to detect the involved reactive species during the photocatalysis process on a JEOL JES‐X320 spectrometer equipped with a microwave power of 10.00 mW and a frequency of 9.15 GHz. The EPR test was carried out in aqueous dispersion and methanol dispersion for DMPO‐•OH and DMPO‐•O_2_^−^ detection, respectively, using 5,5‐dimethyl‐1‐pyrroline N‐oxide (DMPO) as the spin‐trapping agent. Prior to each measurement, 30 mg of BiVO_4_‐Au‐Cu_2_O NSs was dispersed in 10 mL water or methanol. Meanwhile, DMPO solutions were also prepared with a concentration of 400 mm in water or methanol. For the test, 900 µL of the BiVO_4_‐Au‐Cu_2_O NSs suspension and 100 µL of the DMPO solution were mixed under ultrasonication and then transferred into a suprasil EPR tube. The tests were first conducted in the dark at room temperature. Afterward, the suspension was irradiated by a 300 W xenon lamp equipped with a long‐pass filter of 420 nm for 5 min and the EPR measurements were repeated. Photoelectrochemical (PEC) measurements were performed by a standard three‐electrode cell with a CHI 660E electrochemical workstation (CH Instruments). An Ag/AgCl electrode (3 M KCl) and a Pt wire acted as the reference and counter electrode, respectively. For preparing the working electrode, 0.5 g of photocatalysts was dispersed into Nafion solution of ethanol. The suspension was then drop‐casted onto a F‐doped tin oxide (FTO) glass. After natural drying, epoxy resin glue was used to insulate the uncoated part of the FTO glass. Prior to the measurement, the electrolyte solution (0.1 M of Na_2_SO_3_) was purged with N_2_ for 30 min to remove the dissolved O_2_. The PEC measurements were performed under the irradiation of a solar simulator (Newport, 100 mW cm^−2^) with a bias of 0.2 V versus Ag/AgCl. Nyquist plots were obtained at a bias of 0.2 V in the frequency range of 100 mHz to 100 kHz.

### Photocatalytic Activity Measurements and Trapping Experiment

The photocatalytic activities of the as‐prepared photocatalysts were investigated by monitoring Tc degradation in aqueous solution. Typically, 50 mL of Tc solution (30 mg L^−1^) was first mixed with 50 mg of the photocatalysts at a constant temperature of 20 °C. Prior to the photocatalytic reaction, the suspension was vigorously stirred for 30 min in the dark to ensure the adsorption‐desorption equilibrium between the photocatalysts and Tc solution. The photocatalytic reaction was carried out under visible light illumination (λ ≥ 420 nm) generated by a 300 W Xenon lamp with a UV cut‐off filter. At different time intervals, 1 mL of the solution was collected and centrifuged to separate the photocatalysts from the Tc solution. The obtained liquid supernatant was then analyzed using a UV–vis spectrometer to measure the concentration of residual Tc. The separated photocatalysts were then re‐collected and thoroughly washed by DI water for reusability test. The cyclic stability of the photocatalysts was assessed by monitoring the photoactivity of the re‐collected photocatalysts in the same Tc solution for another four runs. Trapping experiments were carried out to detect the active species involved in the photocatalysis process using different specific scavengers during the photocatalysis process. In this study, t‐BuOH (1 mm), BQ (1 mM), and methanol (1 mM) served as scavengers to capture hydroxyl radicals (•OH), superoxide radicals (•O_2_^−^) and holes, respectively.

### Photocatalytic Hydrogen Evolution (HER) Measurements

The photocatalytic hydrogen (H_2_) evolution of the photocatalysts was performed in a 500‐mL Pyrex top‐irradiation reactor with a quartz cover. Specifically, 50 mg of photocatalysts was dispersed in 50 mL of NaSO_3_ solution (0.5 mM) through 10 min ultrasonication. The suspension was then sealed and vacuumed under magnetic stirring for 30 min to remove the air and the dissolved O_2_ in water. The HER reaction took place under the full spectrum of a 300 W Xenon lamp at a constant temperature of 20 °C. The H_2_ evolution volumes were measured by gas chromatography (GC, 7890B, Agilent Technologies) equipped with a thermal conductivity detector.

### Analytical Measurement and Transformation Products Identification

The possible degradation pathway of Tc was identified by investigating the transformation products (TPs) of Tc during the photocatalysis process with a Nexera UHPLC system (Shimadzu, Columbia, MD). Specifically, 1 µL of the degradative Tc solution was injected into a Phenomenex Kinetex C18 column at the column temperature of 40 °C. Water with 0.1% formic acid (A) and acetonitrile with 0.1% formic acid (B) was applied as the mobile phase at a flow rate of 0.3 mL min^−1^. A linear gradient was used starting with 97% (A) for one minute, changing to 50% (A) for 15 min and then 15% (A) for 18 min. The Data were acquired in information‐dependent acquisition (IDA) mode, with time‐of‐flight mass spectrometry (TOF‐MS) survey acquisition.

### Antibacterial Activity Measurement

The Tc solution after photocatalytic treatment by the optimal BiVO_4_‐Au‐Cu_2_O NSs sample was evaluated in terms of the growth inhibition of *Escherichia coli* (*E. coli*), JM 103, by using the optical density method. Specifically, 5 mL of Tc solution (30 mg L^−1^) after photocatalytic treatment for 1, 2, 3, and 6 h, respectively, was mixed with 5 mL of lysogeny broth (LB) solution. Subsequently, 1 mL of *E. coli* strains (≈1.5 × 10^8^ CFU mL^−1^) were added and incubated at 37 °C for 4 h. The absorbance at the wavelength of 600 nm was then measured by a UV–vis spectrophotometer, which served as an indicator for bacterial growth. The initial Tc solution (untreated) and DI water were mixed with LB under sterile conditions as control samples. Two parallel experiments were conducted for each sample. Afterward, all the *E. coli* solutions were incubated at 37 °C for 2, 4, and 24 h, and the absorbances were then detected.

## Conflict of Interest

The authors declare no conflict of interest.

## Author Contributions

C.W. performed conceptualization, methodology, formal analysis, investigation, data curation, visualization, wrote the original draft, and provided resources. A.M., Q.Z., and D.M. performed conceptualization, methodology, formal analysis, supervision, visualization, wrote, reviewed, and edited the draft. Y.W. provided resources. M.C. performed conceptualization, supervision, wrote, reviewed, and edited the draft.

## Supporting information

Supporting Information

## Data Availability

The data that support the findings of this study are available from the corresponding author upon reasonable request.
