# Supplementary material for: Construction of Ultrathin BiVO4‐Au‐Cu2O Nanosheets with Multiple Charge Transfer Paths for Effective Visible‐Light‐Driven Photocatalytic Degradation of Tetracycline
Source: Small Methods. 2024 Jun 10;9(2):2301804. doi: 10.1002/smtd.202301804 (PMC11843413; doi:10.1002/smtd.202301804)
Supplement: Supplementary file 1 — Supporting Information [file SMTD-9-2301804-s001.docx]

**Supporting Information**

**Construction of** **Ultrathin BiVO_4_-Au-Cu_2_O Nanosheets with Multiple Charge Transfer Paths for Effective Visible-Light-Driven Photocatalytic Degradation of Tetracycline**

**Chen Wang^a^, Amir Mirzaei^a^, Yong Wang^a^, Mohamed Chaker^a^, Qingzhe Zhang^b,c,*^, Dongling Ma^a,*^**

*^a^ Institut National de la Recherche Scientifique (INRS), Centre Énergie Materiaux et Télécommunications, 1650 Boulevard Lionel-Boulet, Varennes, Québec, J3X 1P7, Canada*

*^b^ Shandong Key Laboratory of Environmental Processes and Health, School of Environmental Science and Engineering, Shandong University, Qingdao 266237, China*

*^c^ Shenzhen Research Institute of Shandong University, Shenzhen 518057, China*


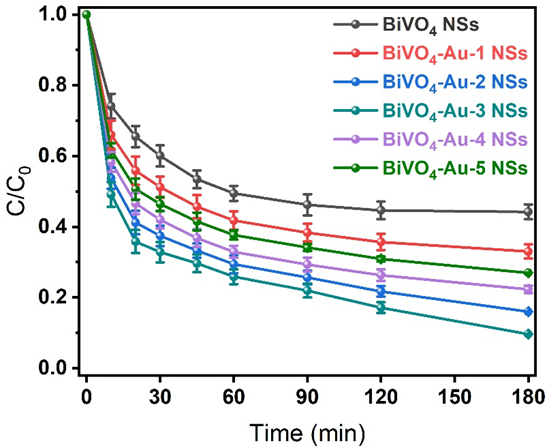


**Figure S1**. The plots of (C/C_0_) vs reaction time for Tc (30 mg/L) in the presence of BiVO_4_-Au NSs with different amounts of Au NPs under λ > 420 nm light irradiation. The measurements were repeated for three times, and the average values were used as the data with the error bars shown in the picture.


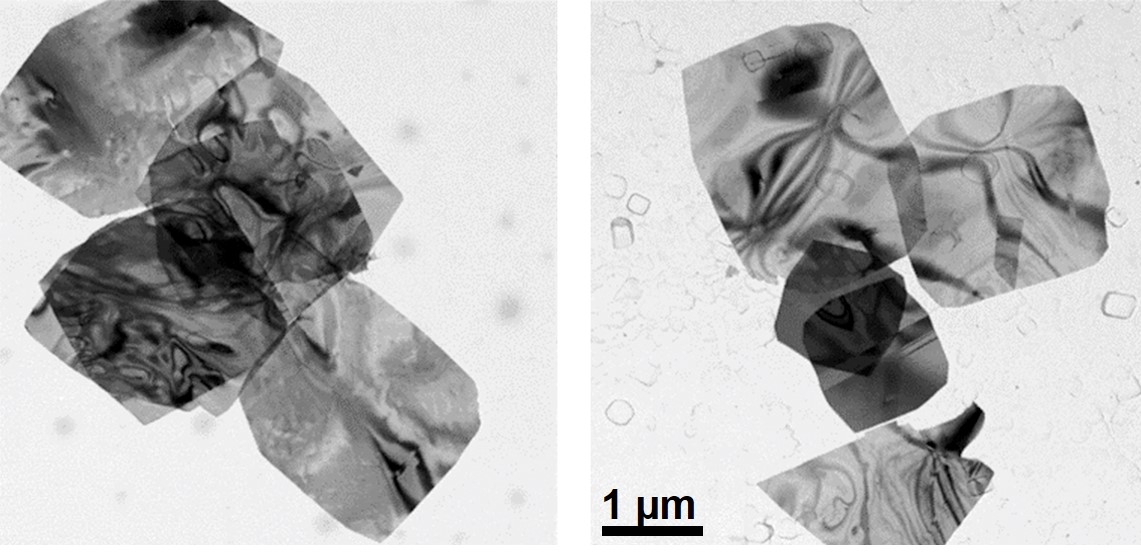


**Figure S2**. TEM images of as-prepared BiVO_4_ NSs before ligand exchange.


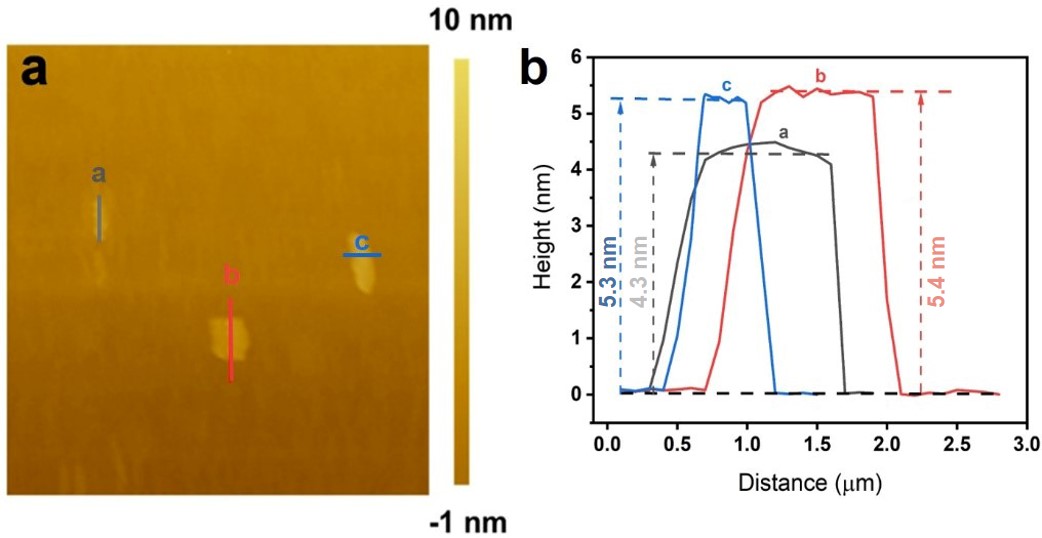


**Figure S3**. (**a**) Tapping mode AFM topographical images of the BiVO_4_ NSs and (**b**) corresponding height profiles of the BiVO_4_ NSs along the gray, red and blue lines in (**a**).


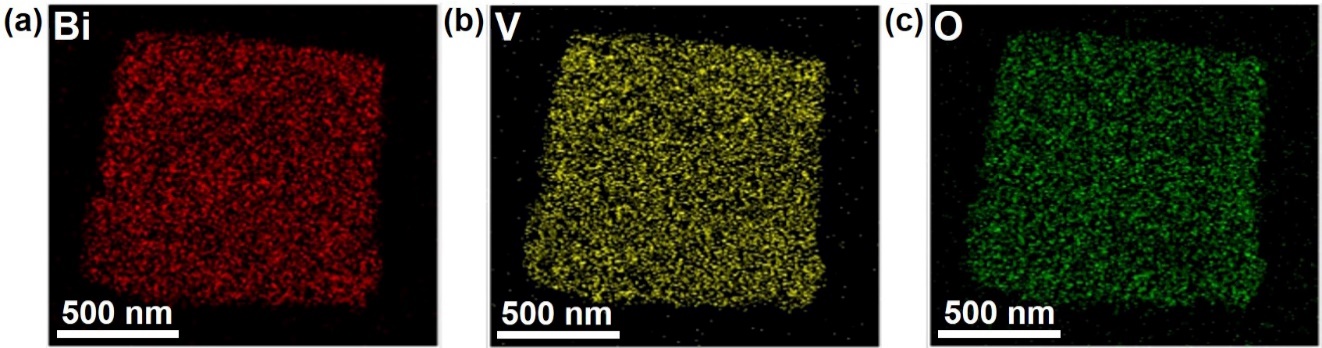


**Figure S4**. EDS mapping images of the elements of **(a)** Bi, **(b)** V and **(c)** O in the BiVO_4_ NSs.


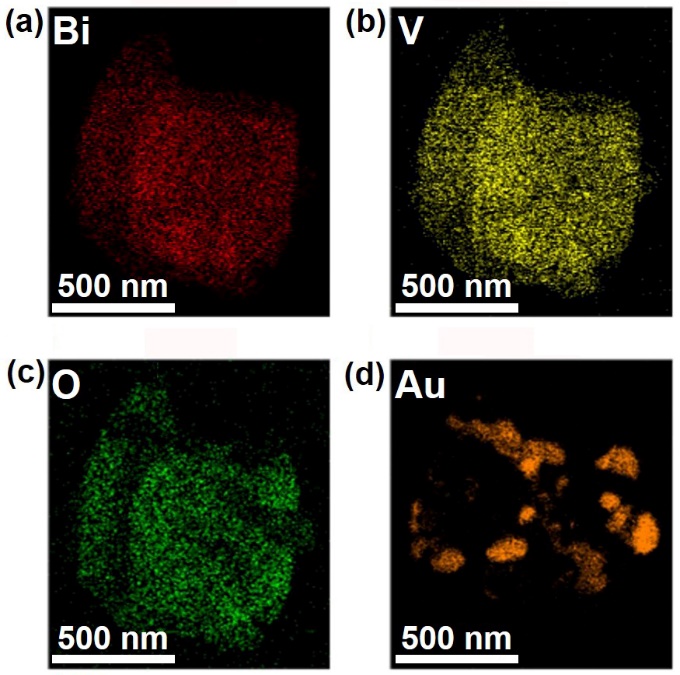


**Figure S5**. EDS mapping images of the elements of **(a)** Bi, **(b)** V, **(c)** O and **(d)** Au in the BiVO_4_-Au NSs.


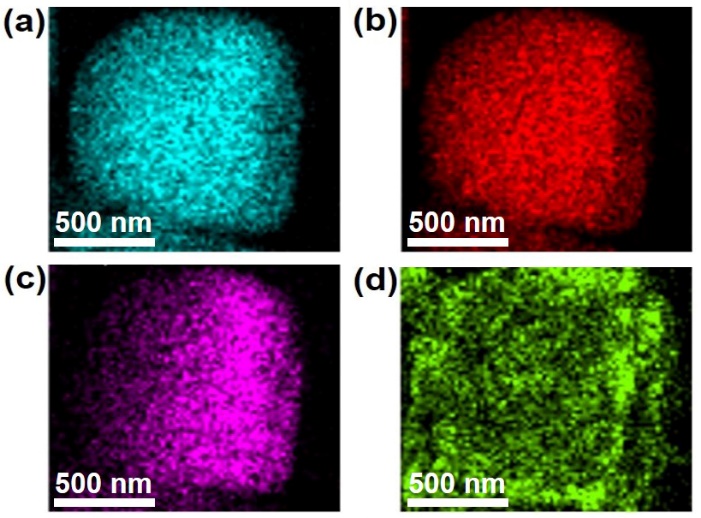


**Figure S6**. EDS mapping images of the elements of **(a)** Bi, **(b)** V, **(c)** O and **(d)** Cu in the BiVO_4_-Cu_2_O NSs.


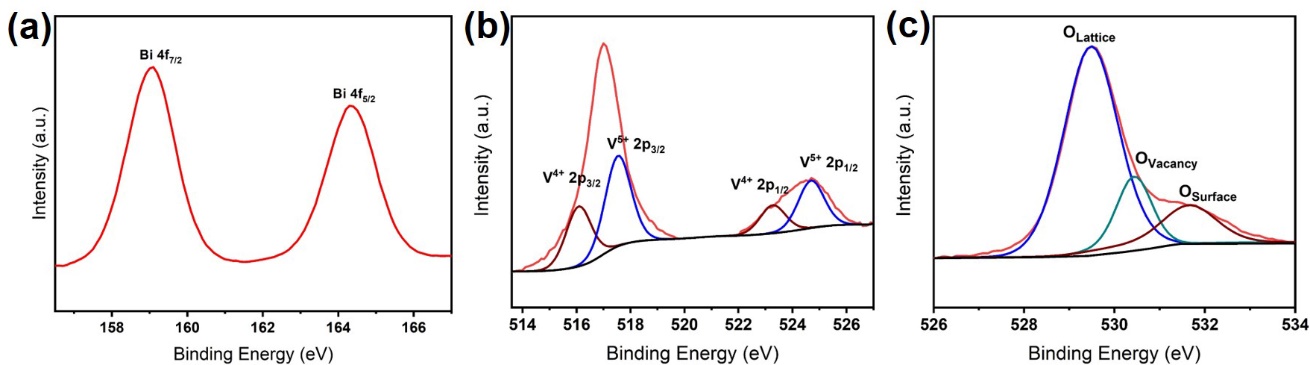


**Figure S7**. High-resolution XPS spectra of the elements of **(a)** Bi, **(b)** V and **(c)** O in the BiVO_4_ NSs.


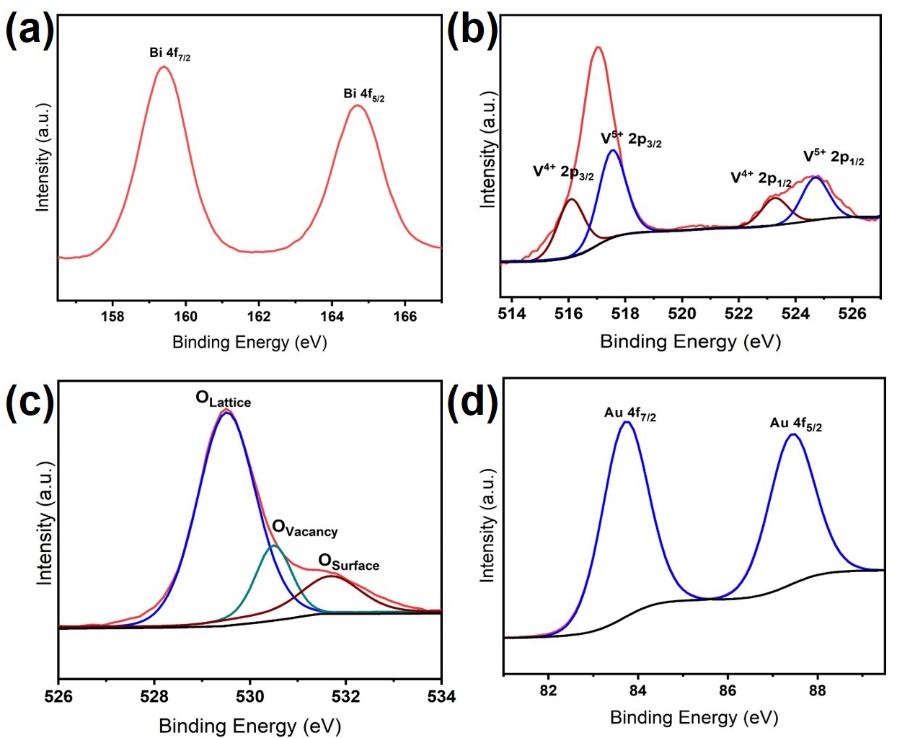


**Figure S8**. High-resolution XPS spectra of the elements of **(a)** Bi, **(b)** V **(c)** O and **(d)** Au in the BiVO_4_-Au NSs.


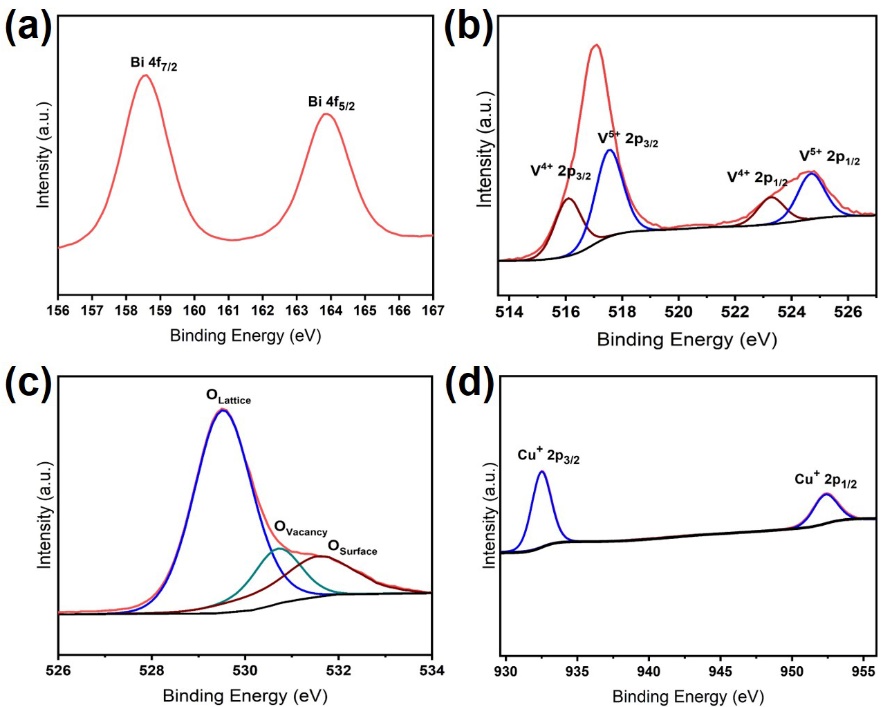


**Figure S9**. High-resolution XPS spectra of the elements of **(a)** Bi, **(b)** V and **(c)** O and **(d)** Cu in the BiVO_4_-Cu_2_O NSs.


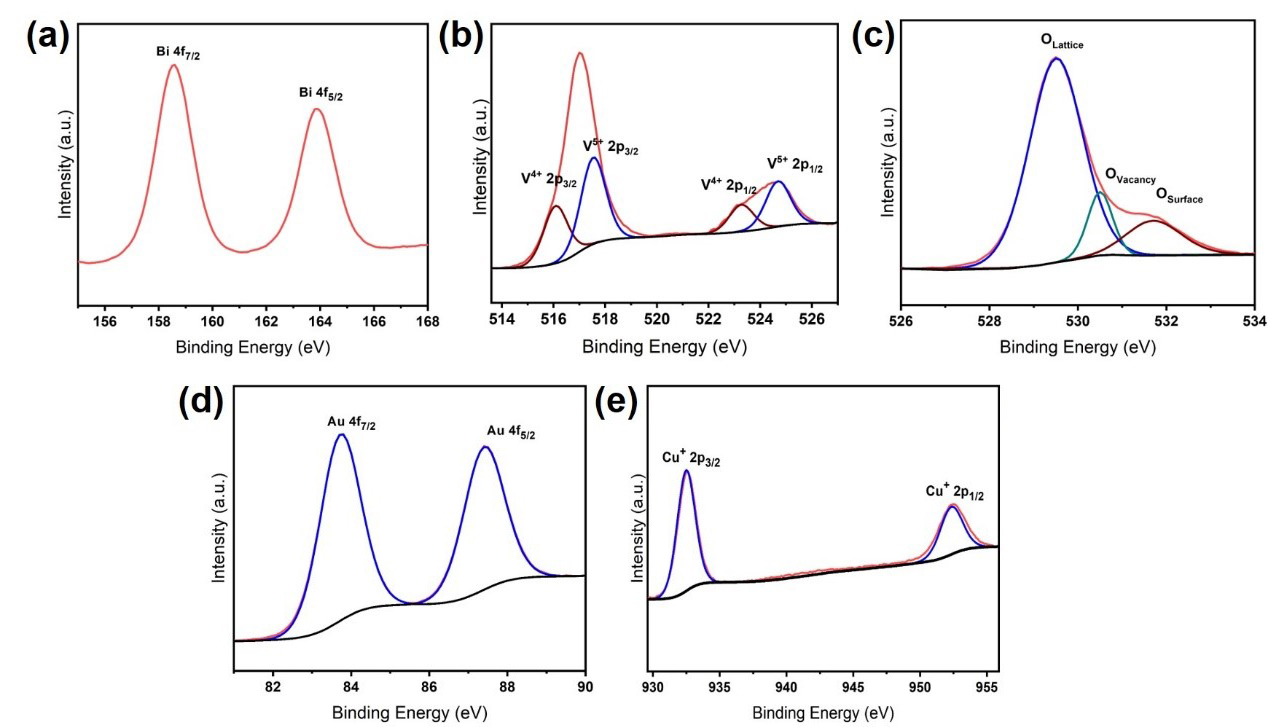


**Figure S10**. High-resolution XPS spectra of the elements of **(a)** Bi, **(b)** V, **(c)** O, **(d)** Au and **(e)** Cu in the BiVO_4_-Au-Cu_2_O NSs.


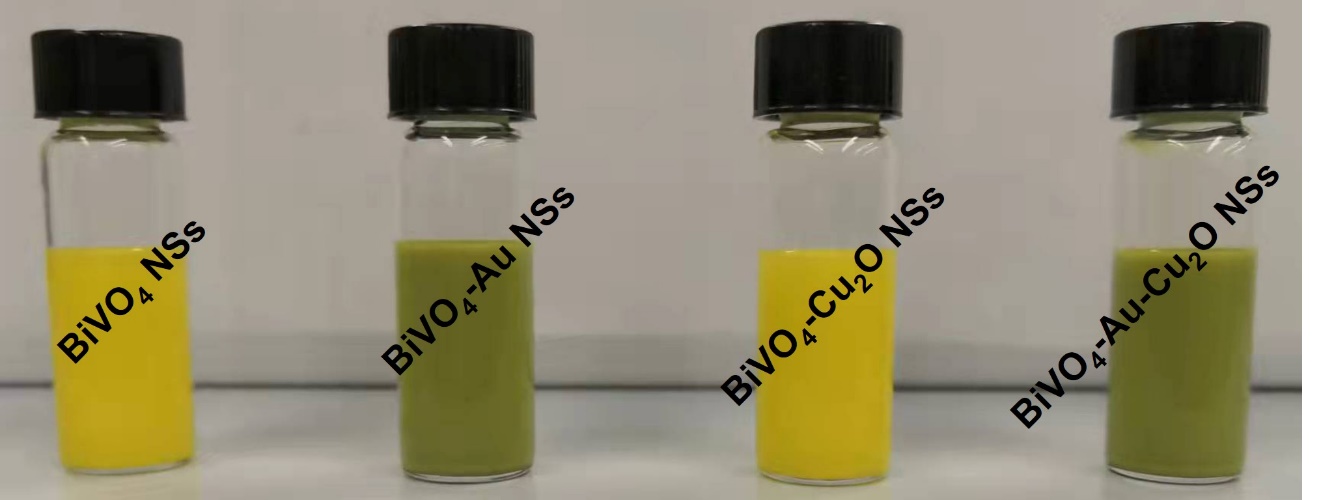


**Figure S11.** Photographs of the BiVO_4_ NSs, BiVO_4_-Au NSs, BiVO_4_-Cu_2_O NSs and BiVO_4_-Au-Cu_2_O NSs, respectively.

**Table S1**. The average lifetime and lifetime components of the BiVO_4_ NSs and BiVO_4_-based hybrids NSs.

| **Sample** | **Lifetime (ns)** | **τ_1_** | **τ_2_** | **B_1_** | **B_2_** |
| --- | --- | --- | --- | --- | --- |
| **BiVO_4_ NSs** | 614.81 | 801.61 | 58.99 | 19.21 | 87.73 |
| **BiVO_4_-Au NSs** | 512.88 | 679.41 | 50.91 | 20.49 | 98.94 |
| **BiVO_4_-Cu_2_O NSs** | 552.36 | 730.31 | 55.19 | 19.73 | 93.45 |
| **BiVO_4_-Au-Cu_2_O NSs** | 474.38 | 626.98 | 40.58 | 24.28 | 131.96 |


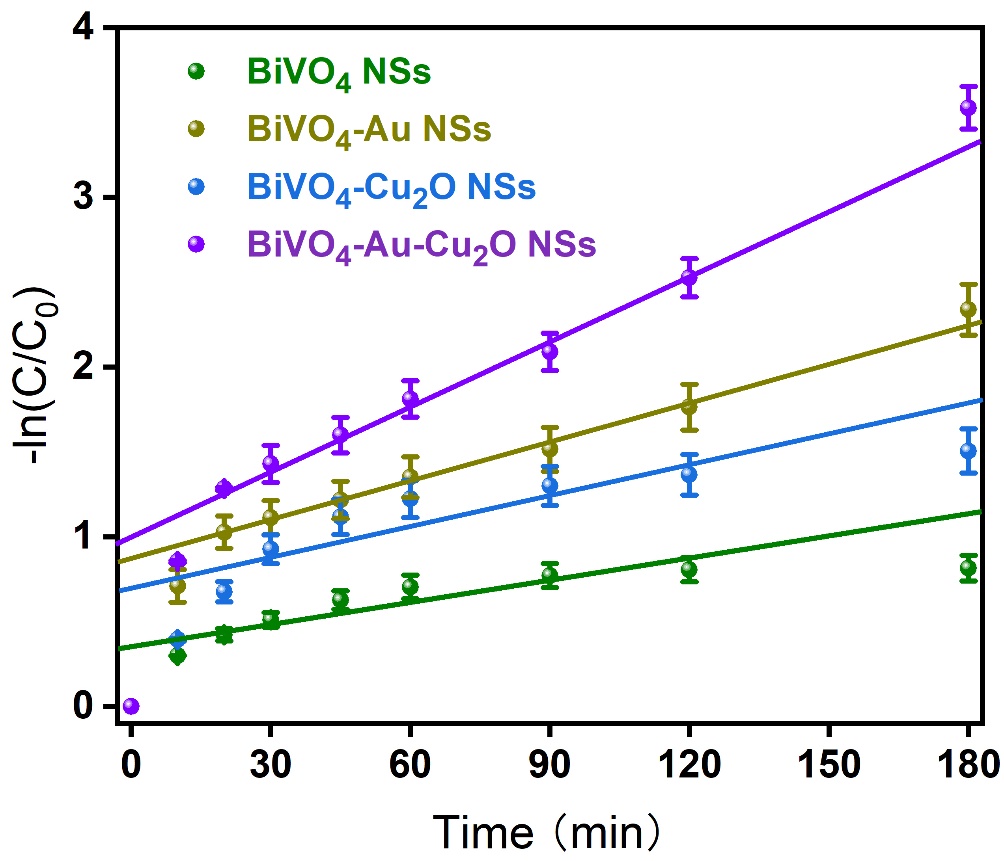


**Figure S12.** Plots of -ln(C/C_0_) vs reaction time of Tc solution in the presence of the BiVO_4_ NSs, BiVO_4_-Au NSs, BiVO_4_-Cu_2_O NSs and BiVO_4_-Au-Cu_2_O NSs under visible light irradiation (λ > 420 nm). The measurements were repeated for three times, and both the average values and error bars were included.


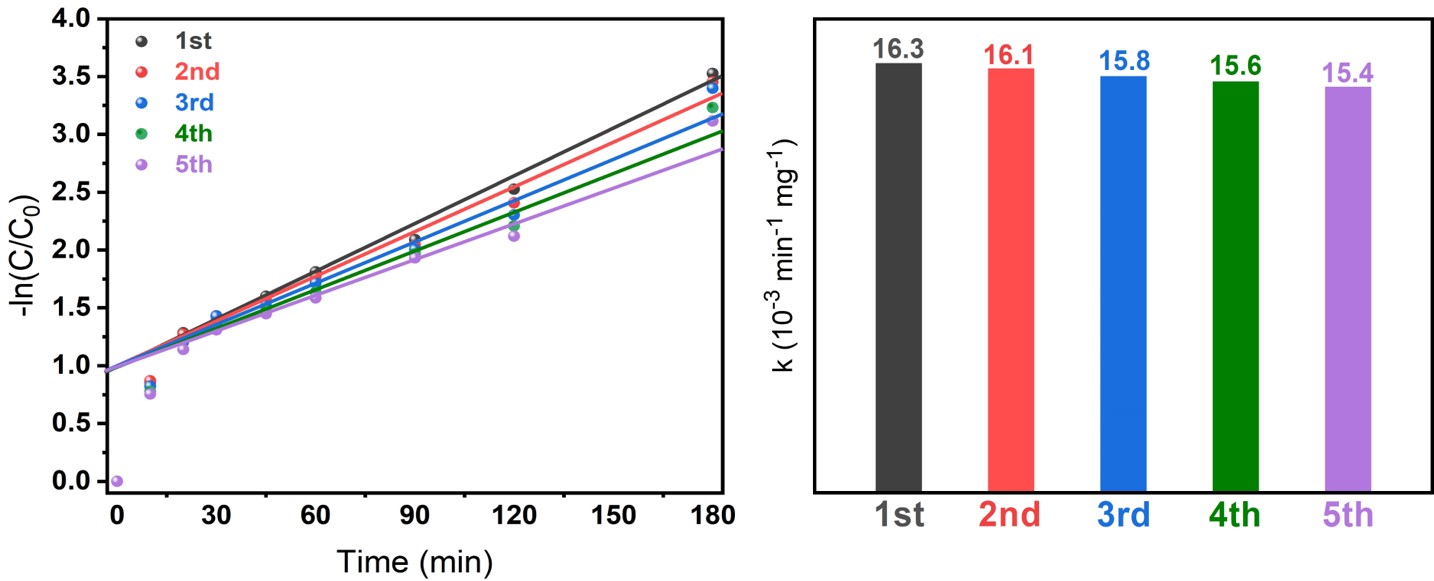


**Figure S13.** **(a)** Plots of -ln(C/C_0_) vs reaction time of Tc solution in the presence of the BiVO_4_-Au-Cu_2_O NSs and **(b)** corresponding degradation rate constants in five successive cycles under λ > 420 nm.

**Figure S14.** Concentration evolution of the Tc degradation products during visible-driven photocatalytic degradation by using BiVO_4_-Au-Cu_2_O NSs as the photocatalyst.


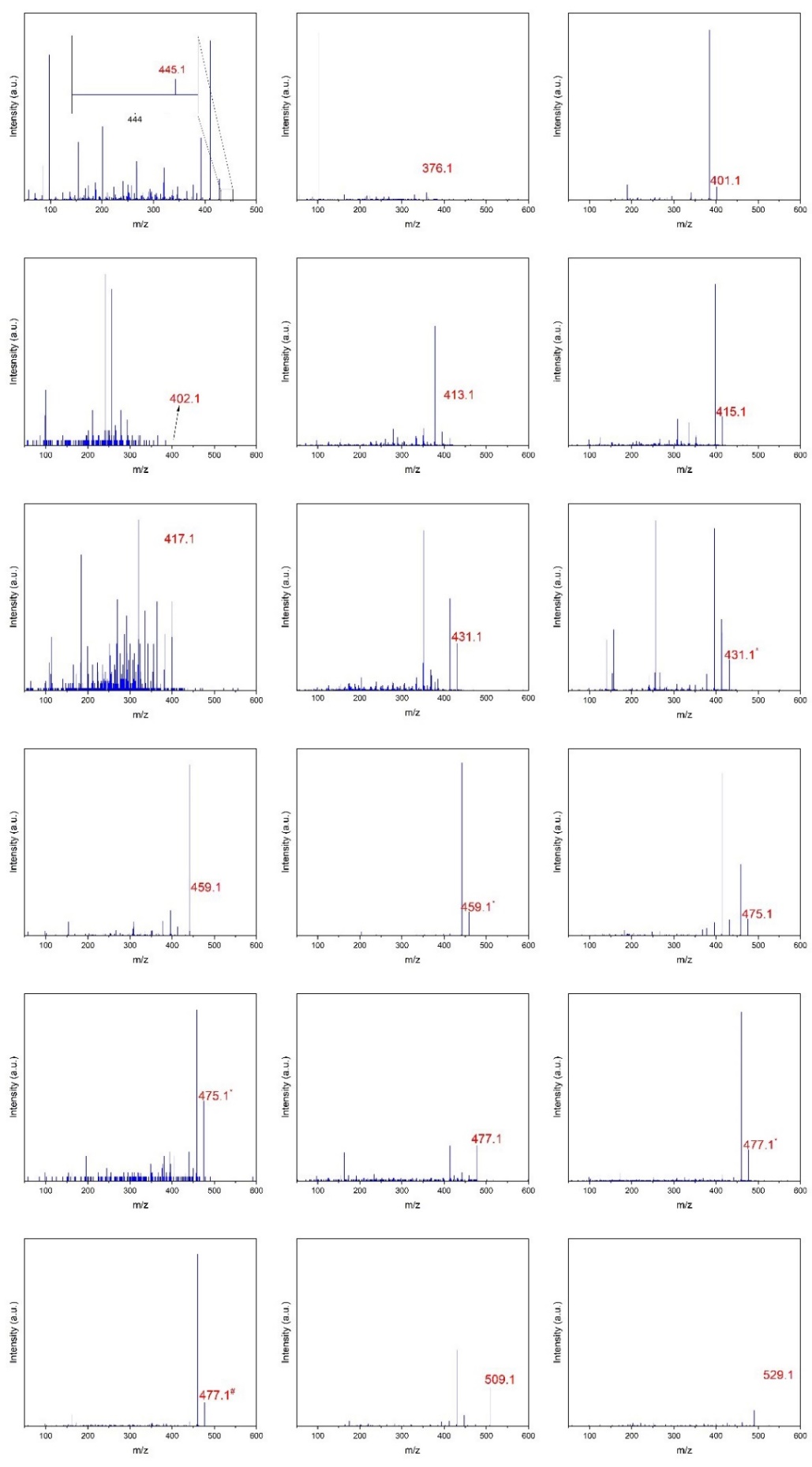


**Figure S15.** MS/MS spectra of the Tc and the detected DPs. The positive ion ESI MS/MS spectra were used to identify the DPs generated during the photocatalytic degradation of Tc under λ > 420 nm light irradiation.


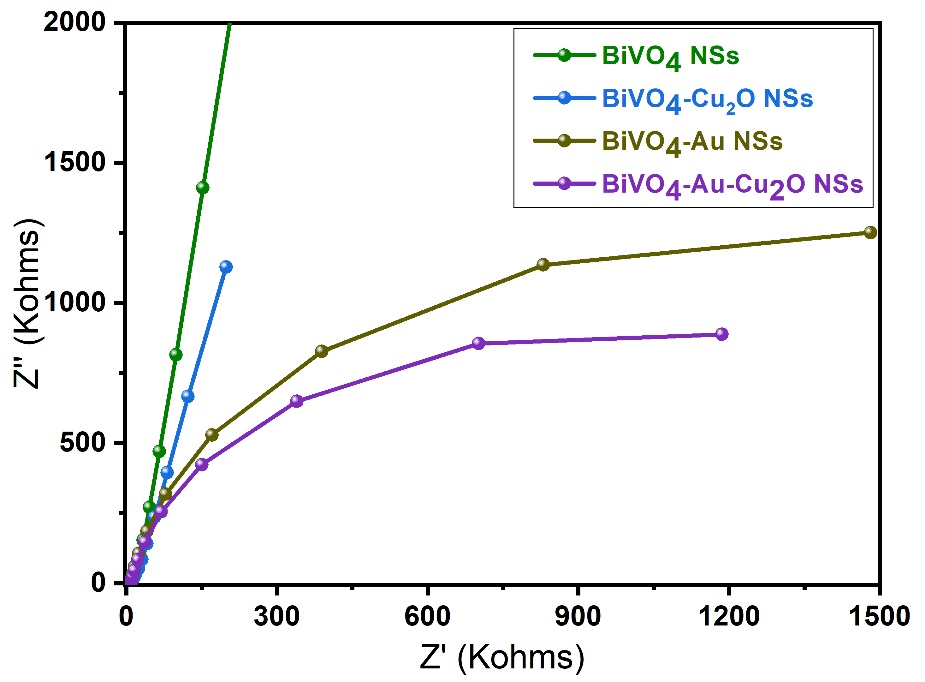


**Figure S16.** EIS Nyquist plots under simulated solar light irradiation of the BiVO_4_ NSs, BiVO_4_-Au NSs, BiVO_4_-Cu_2_O NSs and BiVO_4_-Au-Cu_2_O NSs.


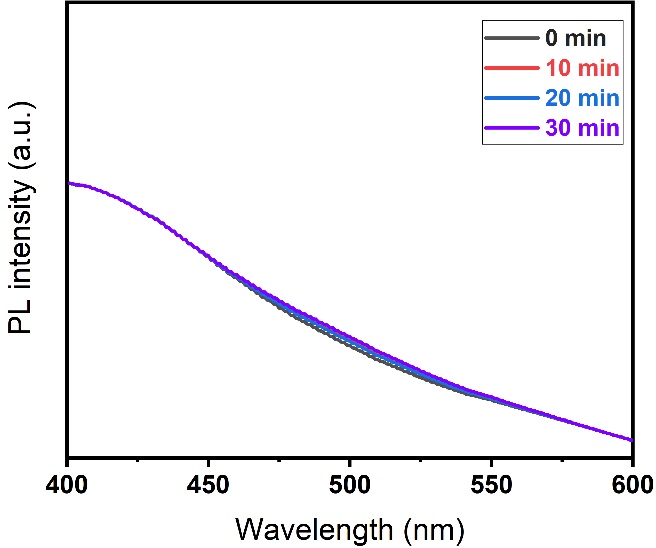


**Figure S17.** PL spectral change of the coumarin solution in the presence of the BiVO_4_-Cu_2_O NSs under visible light illumination.


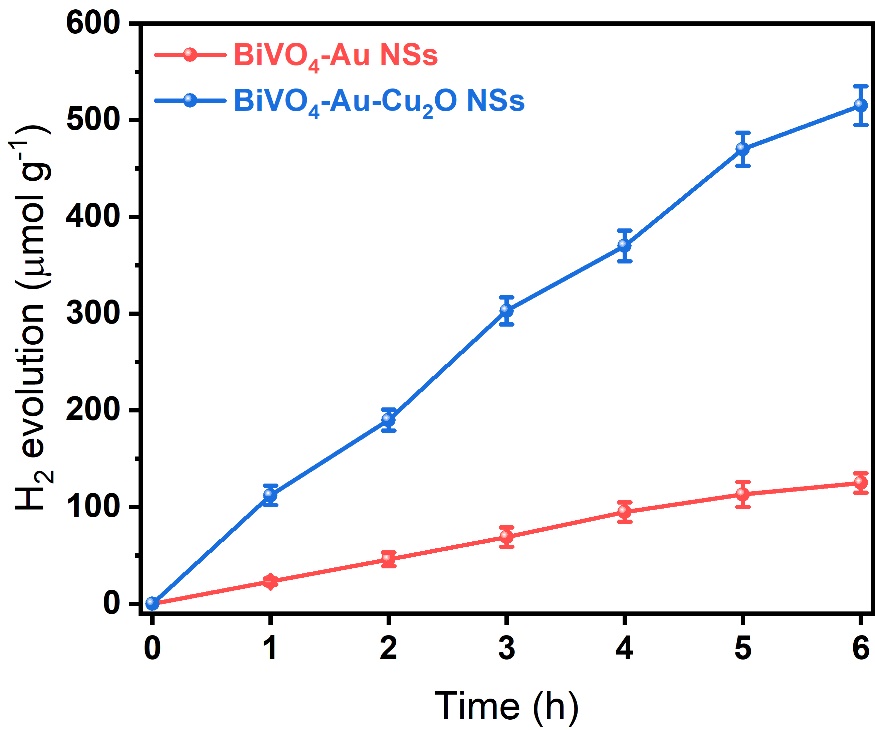


**Figure S18.** Hydrogen evolution versus time of the BiVO_4_-Au NSs and BiVO_4_-Au-Cu_2_O NSs. The test was carried out under the full solar spectrum in the solution of NaSO_3_ solution (0.5 mM). The measurements were repeated for three times, and both the average values and error bars are presented.
